# Higher temperature variability increases the impact of *Batrachochytrium dendrobatidis* and shifts interspecific interactions in tadpole mesocosms

**DOI:** 10.1002/ece3.369

**Published:** 2012-08-29

**Authors:** Phineas T Hamilton, Jean ML Richardson, Purnima Govindarajulu, Bradley R Anholt

**Affiliations:** 1 Department of Biology, University of VictoriaPO Box 3020, STN CSC, Victoria, British Columbia, V8W 3N5, Canada; 2 British Columbia Ministry of EnvironmentPO Box 9338, STN Prov Govt, Victoria, British Columbia, V8W 9M1, Canada; 3 Bamfield Marine Sciences CentreBamfield, British Columbia, V0R 1B0, Canada

**Keywords:** Chytridiomycosis, climate change, competition, mesocosm, tadpole, temperature

## Abstract

The emergence of amphibian chytridiomycosis, caused by the fungus *Batrachochytrium dendrobatidis* (Bd) has led to the decline and extinction of numerous amphibian species. Multiple studies have observed links between climatic factors and amphibian declines apparently caused by Bd. Using outdoor experimental mesocosms, we tested the response of red-legged frog (*Rana aurora*) tadpoles to increased variation in temperature, a component of climate linked to amphibian declines, and Bd exposure. We included tadpoles of a sympatric competitor species, Pacific chorus frog (*Pseudacris regilla*), in a fully factorial design to test the effects of Bd and temperature on interspecific interactions. We found that higher variation in temperature had numerous effects in mesocosms, including interacting with Bd presence to decrease the condition of *R. aurora*, shifting the relative performance of competing *P. regilla* and *R. aurora*, and accelerating the development of *P. regilla* relative to *R. aurora*. Our results demonstrate that increased variation in temperature can affect amphibians in multiple ways that will be contingent on ecological context, including the presence of Bd and competing species.

## Introduction

Major predicted consequences of global climate change include the increasing frequency, severity, and geographic range of infectious disease (Patz et al. 2005; Rohr et al. 2011). Evidence from a variety of systems predicts that climate change will influence disease dynamics (Patz et al. 2005; Paaijmans et al. 2010; Rohr et al. 2011; Lambrechts et al. 2011), or has already begun to do so (Rohr and Raffel 2010). Nonetheless, many researchers have pointed out that predicting the consequences of climate change in disease systems will not be straightforward (Patz et al. 2005; Lafferty 2009; Rohr et al. 2011). Many examples of interactions between disease and climate change remain correlational and confounded by multiple factors (Rohr et al. 2011), making the study of specific climatic effects on disease in controlled experiments increasingly relevant.

The dynamics of the disease chytridiomycosis in amphibians may be an example of a wildlife disease that is affected by climate (Rohr and Raffel 2010). First characterized in 1998 (Berger et al. 1998; Longcore et al. 1999), chytridiomycosis has since been implicated as the cause of broad-scale declines and extinctions of numerous amphibian species (Skerratt et al. 2007; Fisher et al. 2009a). Chytridiomycosis is caused by infection with the chytrid fungus *Batrachochytrium dendrobatidis* (Bd) (Berger et al. 1998; Longcore et al. 1999). Bd has been shown to be exceptionally virulent in some circumstances, having apparently driven to extinction multiple amphibian species (reviewed by Skerratt et al. 2007).

Numerous studies have demonstrated that the prevalence of chytridiomycosis can be linked to broad-scale climatic factors, such as cooler temperatures due to altitude, latitude or seasonality (e.g., Berger et al. 2004; Kriger and Hero 2007; Kriger et al. 2007). Others have also suggested that interactions with climate change may have driven the emergence of the disease or the severity of outbreaks (Pounds et al. 2006; Bosch et al. 2007; Rohr and Raffel 2010). Links between increasing mean temperature and chytridiomycosis present somewhat of a paradox, however, as Bd is intolerant of warm temperatures, with decreased growth or death above 28°C (Piotrowski et al. 2004). Experimentally, some amphibians can be cured of Bd infection by exposure to elevated temperatures (e.g., Woodhams et al. 2003), and warmer local climates have even been proposed as refuges from the disease (Puschendorf et al. 2006, 2009). However, a recent reanalysis of declines in Central America has suggested that increasing variability in temperature, including increased diurnal temperature range, rather than increasing mean temperature, has contributed to declines linked to disease (Rohr and Raffel 2010).

Increased variation in temperature is increasingly linked to effects on disease dynamics. In addition to its apparent role in amphibian declines (Rohr and Raffel 2010), temperature fluctuation influences the dynamics of human malaria and dengue virus transmission by mosquitos (Paaijmans et al. 2010; Lambrechts et al. 2011). Although physiological studies have consistently demonstrated that higher mean temperatures should benefit amphibians in the amphibian-Bd host-pathogen relationship (e.g., Berger et al. 2004), there is direct evidence that higher variation in temperature can impair amphibian immune function (Raffel et al. 2006), providing a logical proximate mechanism for climate to exacerbate disease in this system. The typically higher daily variation in temperature observed at higher elevations may also contribute to Bd dynamics (Woodhams et al. 2008; Rohr and Raffel 2010).

To evaluate the role of daily temperature variability (hereafter standard deviation of temperature: SDT) in the amphibian-Bd host-pathogen system, we exposed red-legged frog (*Rana aurora*) tadpoles to Bd in outdoor mesocosms and to high SDT and low SDT treatments. Notably, the effects of climate on disease are predicted be contingent on the ecological community in which species are embedded (Lafferty 2009); both parasites (e.g. Jaenike 1995) and climate (e.g. Walther et al. 2002) individually can also influence the strength and outcome of interspecific interactions. As these effects will depend on ecological context, we included tadpoles of Pacific chorus frogs (*Pseudacris regilla*), a sympatric species, in experimental mesocosms with all treatments crossed. Previous study has demonstrated competition between *P. regilla* and *R. aurora* for food resources in mesocosms (Govindarajulu 2004); *P. regilla* has also recently been suggested to serve as a largely asymptomatic host of Bd that can effectively disseminate infection (Reeder et al. 2012), raising the possibility of more complex Bd infection dynamics in the presence of *P. regilla*.

Our experimental design allowed us to test multiple hypotheses. Most importantly: (1) that increased daily variation in temperature increases the transmission and effects of Bd in *R. aurora* tadpoles, (2) that increased temperature variance influences the relative performance of *R*. *aurora* and *P. regilla*, and (3) that higher temperature variance and Bd can interact to affect the performance heterospecific competitors in mesocosms.

## Methods

### Mesocosm system

We tested the effects of temperature variability (SDT), Bd, and the competitor *P. regilla* in outdoor mesocosms containing *R. aurora* using a factorial design (Table 1). This yielded a total of eight treatment combinations, which we replicated six times each in a complete block design (*N* = 48). We established mesocosms in large circular polyethylene “cattle tanks,” (1.6 m diameter by 0.45 m deep), on the grounds of the University of Victoria, Canada. This study was run concurrently with another study on-site, with treatments from the two experiments randomly interspersed. This allowed the use of a subset of tanks (*n* = 12) in both analyses (Hamilton et al. 2012).

**Table 1:** Experimental treatments. There are a total of eight treatments, each replicated six times in a factorial design. Each tank contained either 60 *Rana aurora* or 30 *R. aurora* and 30 *Pseudacris regilla*

	Bd absent		Bd present	
		
	*R. aurora*	*P. regilla*	*R. aurora*	*P. regilla*
Low SDT	60	0	60	0
	30	30	30	30
High SDT	60	0	60	0
	30	30	30	30

Bd, *Batrachochytrium dendrobatidis*.

Prior to the start of the experiment, we disinfected mesocosm tanks with 10% household bleach solution, rinsed them, and allowed them to air dry. We filled tanks with ∼600 L of tapwater on 12 April 2009. After 3 days, we added 1 kg of autoclaved leaf litter and a 0.5 mL aliquot of concentrated *Chlorella vulgaris* algae from a lab culture. On 7 May, and twice more within that week, we added lab-reared *Daphnia magna* and *Daphnia pulex* to ponds. We included *Daphnia* as they are typically included in experimental mesocosms as integral parts of functioning ponds. Additionally, *Daphnia* have been shown to be important to the growth of *R. aurora* in mesocosms in recent experiments (Hamilton et al. 2012). During this experiment we quantified the number *Daphnia* in each tank using previously described methodology (Hamilton et al. 2012), which allowed us to account for potential effects of *Daphnia* on the experimental factors examined herein.

Also, on 7 May, and continuing every 10 days throughout the experiment, we added NaNO_3_ and K_3_PO_4_ at an atomic ratio of 40:1 N:P to a concentration of 33 μg P L^−1^ to discourage the growth of toxic cyanobacteria (Anholt 1994). Although other studies have typically seeded mesocosms with collections from natural ponds, we avoided this approach, as we were concerned with inadvertently adding Bd to mesocosms (Walker et al. 2007). Throughout the experiment, we kept tanks securely covered with 40% shade cloth covers affixed to weighted rims.

### Experimental treatments

#### Rana aurora

We collected *R. aurora* egg masses from Bamfield, BC (48º58′46′′N, 125º7′16′′W) and Port Renfrew, BC (46º29′43′′N, 124º16′18′′W) in March 2009, and returned them to the University of Victoria for rearing. We mixed egg masses and hatched eggs in plastic wading pools filled with aged tapwater (hereafter “rearing tanks”). When tadpoles had reached the actively foraging Gosner stage 25 (Gosner 1960), we provided crushed Hagen® (Baie d'Urfe, Quebec) rabbit pellets for food. When all hatchlings had reached stage 25–26, we transferred tadpoles to mesocosm treatments. We placed 55 tadpoles in treatment tanks not containing *P. regilla* and 25 in those with *P. regilla*.

#### Pseudacris regilla

We collected *P. regilla* egg masses from near Sooke, BC (48º20′13′′N, 128º38′37′′W) in April 2009 and reared them similar to *R. aurora*. Once tadpoles had reached Gosner stage 25, we transferred 30 *P. regilla* to each *P. regilla* present in the treatment tank. We chose a substitutive design, holding overall tadpole density constant, as pathogen transmission is well known to be affected by the density of susceptible hosts, and we did not wish to confound this aspect of our study (McCallum et al. 2001). Nonetheless, to accommodate potential intraspecific density-dependent effects, we included the total number of *R. aurora* surviving until the end of the experiment as a covariate in our statistical models (see below).

#### Temperature

**Figure 1:**
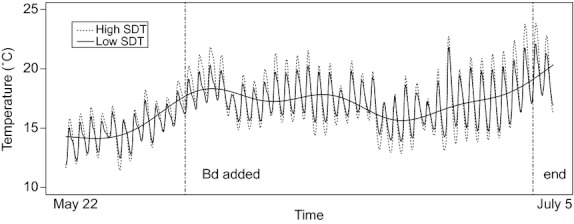
The mean temperature at each recorded time point (averaged across tanks) in low and high standard deviation of temperature treatments; the central line represents the smoothed fit of a generalized additive model.

We manipulated the temperature in tanks by burying half of the mesocosm tanks to a depth of 30 cm in the ground. We chose this approach to cool tanks as prior experiments on-site suggested that mesocosm temperatures would exceed the thermal tolerance of Bd if tanks were heated rather than cooled (P. Govindarajulu unpubl. data). We measured the effect of burying on temperature using iButton® (Maxim, Inc. Sunnyvale, CA) temperature loggers, sealed in 50 mL centrifuge tubes in the bottom eastern edge of each tank. These loggers recorded temperature every hour throughout the experiment. We calculated average temperature standard deviation (STD) for each tank as the standard deviation of temperature for the tank for each day and averaging it across days for the duration of the experiment. The insulating effect of the ground effectively decreased SDT in buried tanks (mean SDT = 1.25°C) versus unburied tanks (mean SDT = 1.83°C) (Fig. 1, Mann–Whitney *U-*test, *P* < 0.0001). Burying tanks to create a difference in temperature variability also marginally affected mean temperature (16.80°C vs. 16.98°C for buried and unburied tanks respectively; Mann–Whitney *U-*test, *P* = 0.047). In sum, tanks above ground had 47%, or 0.58°C, greater SDT, but only a 1%, or 0.18°C, increase in mean temperature (relative to 0°C). Buried tanks thus represent low SDT treatments and unburied tanks high SDT treatments. Temperature loggers in four tanks (three in low SDT and one in high SDT treatments) failed to record, hence these tanks were excluded from these calculations.

#### Batrachochytrium dendrobatidis

Bd for the experiment was obtained using standard methods to culture a local isolate of Bd from an infected bullfrog (*Lithobates catesbeianus*) tadpole collected in Nanaimo, BC (Longcore et al. 1999). We used a local isolate of Bd because differences in virulence have been observed between strains (e.g., Fisher et al. 2009b), and we desired an ecologically realistic test of the effect of a local isolate on local amphibians.

To infect *R. aurora* tadpoles, we grew and harvested zoospores using established methods (Longcore et al. 1999). To small tanks, holding 15 tadpoles in 12 L of water, we added ∼2 × 10^5^ zoospores every other day. After 8 days, we sacrificed 10 tadpoles at random and tested them for Bd using quantitative PCR (*n* = 7/10 infected; see methods below). We added five of the tadpoles from infection tanks to each Bd-present mesocosm. The probability that no infected tadpoles were added to a Bd-present mesocosm is 0.3^5^ = 0.002, assuming independent probabilities of infection. To each Bd-absent mesocosm we added, five tadpoles directly from the rearing tanks, after using qPCR to confirm these tanks had not become contaminated with Bd (*n* = 0/20 infected). After this addition, all mesocosms had 60 tadpoles total.

### Ending mesocosms

*Pseudacris regilla* began to metamorphose the last week of June, earlier than *R. aurora* tadpoles. We checked tanks daily for *P. regilla* metamorphs and removed and euthanized them to prevent their escape from mesocosms, although they appeared unable to escape lids. Throughout, we euthanized animals with an overdose of buffered MS-222 (tricaine methanesulfonate), transferred them to individual plastic bags, and froze them at −20°C until further analysis.

We ended the experiment when *R. aurora* tadpoles in the majority of tanks began to metamorphose, on 3 July. We dip-netted tadpoles and metamorphs from tanks, and disinfected dip-nets between tanks using 10% bleach. We used this method as tanks were known to contain Bd, hence water could not be directly pumped out prior to disinfection. Dip-netting stirred up debris in tanks and some tadpoles were missed in our initial tadpole collection (mean 0.43 tadpoles per tank). These tadpoles were collected later and included in estimates of tadpole survivorship, but excluded from analyses of tadpole size, as they had spent longer in tanks.

### Data collection and analysis

We tested for effects of treatments on the mass, snout-vent-length (hereafter length), and survivorship of tadpoles (both *R. aurora* and *P. regilla*) in mesocosms. We weighed, measured, and staged (Gosner 1960) frozen tadpoles and metamorphs and recorded survivorship in tanks.

We tested tadpoles for Bd using established DNA extraction methods and quantitative PCR (qPCR) protocols (Boyle et al. 2004) using a StrataGene Mx4000 system (StrataGene, La Jolla, CA). We screened tadpoles by collecting tissue samples using sterile technique to avoid cross-contamination between individuals. From tadpoles (Gosner stage ≤41), we dissected mouthparts and extracted DNA. From metamorphs (Gosner stage >41), we clipped toes from hind feet for testing. We tested all survivors in Bd-exposed tanks (*n* = 893) and up to 10, depending on survivorship, tadpoles from unexposed treatments to confirm the absence of Bd. The majority of samples from one Bd-exposed tank became degraded due to an accidental thaw, after tadpoles had been measured, but prior to DNA extraction. This tank was therefore not tested for Bd.

### *Rana aurora* analyses

*Rana aurora* tadpoles were at various developmental stages at the end of the experiment, but there was no detectable effect of treatments on stage (ANCOVA; *P* > 0.3 for all factors). Thus, to control for different stages when analyzing treatment effects on mass and length, we used generalized additive models (GAMs) to predict the mass and length of tadpoles at metamorphosis (Stage 41), and conducted further analyses on these predicted values (modified from Werner and Anholt 1996). We included all *R. aurora* in the experiment in GAMs simultaneously, then averaged the predicted mass and length for each tank.

We analyzed survivorship using a randomization test of analysis of variance (ANOVA) (Manly 2001), because a preliminary binomial generalized linear model (GLM) showed significant overdispersion. We included the temperature treatment, *P. regilla* presence and Bd presence as predictors and used the proportion of *R. aurora* surviving as the response, using 10,000 reshufflings of the data to generate an empirical distribution of *F*-ratios from which to calculate *P*-values.

To analyze treatment effects on *R. aurora* length, we used analysis of covariance (ANCOVA), with treatments as predictors, and included the number of surviving tadpoles as a covariate to incorporate density-dependent effects. We simplified this full model using the *step*() function in R v. 2.9.2 (http://www.r-project.org), which sequentially removes model parameters if removal results in an improved (lower) AIC score (Venables and Ripley 2002; Crawley 2007). We analyzed tadpole mass similarly, but also incorporated tadpole length as a covariate in the ANCOVA to provide a measure of tadpole body condition (mass while controlling for length, or “fatness,” Garcia-Berthou 2001). We investigated effects of *Daphnia* on tadpoles by including their number in tanks as covariates in appropriate models. We additionally tested *Daphnia* response to experimental treatments (SDT × Bd × *P. regilla*) using ANOVA with number of *Daphnia* per tank as the response variable.

### *Pseudacris regilla* analyses

In contrast with *R. aurora*, *P. regilla* development stage was clearly affected by treatments (see results). For consistency, we also controlled for effects of these stage differences with GAMs. We analyzed length and mass as described for *R. aurora* above, but without *R. aurora* presence as a factor, as *R. aurora* were present in all tanks. Aswe felt that the most interesting comparison of the development rates of *P. regilla* was with respect to *R. aurora* in the same tanks, we subtracted the average development stage of *R. aurora* from that of *P. regilla* in each tank to provide a metric of the differential effects of treatments on the two species. We included early metamorphosing *P. regilla* in this analysis conservatively at Gosner stage 46, and analyzed these differences with ANCOVA, again including surviving *R. aurora* and *Daphnia* numbers as covariates in the model.

### *Batrachochytrium dendrobatidis* transmission

We analyzed the effects of treatments on the proportion of survivors (*R. aurora* and *P. regilla* pooled) infected with Bd at the end of the experiment using a generalized linear model with binomial errors and logit link. All statistical analyses were performed using the statistical software R.

## Results

**Figure 2:**
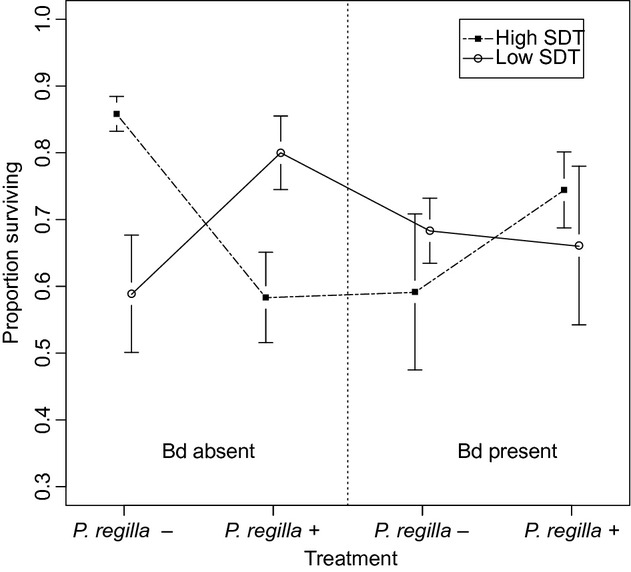
Survivorship of *Rana aurora* in different treatments. Confidence intervals represent treatment mean ± SE. There is a significant interaction between the three experimental factors (temperature, Bd, and *Pseudacris regilla* presence; *P* = 0.004). Survivorship of *P. regilla* was not affected SDT or Bd.

The mean survival of *R. aurora* in the experiment was 69% and was largely unaffected by experimental treatments (ANCOVA *R*^2^ = 0.1). However, there was a significant three-way interaction between experimental factors that affected survival (ANCOVA randomized; *F*_1,40_ = 8.85, *P* = 0.004). In Bd-unexposed treatments, *R. aurora* survival increased with high SDT when *P. regilla* were present. In Bd-exposed treatments *R. aurora* survivorship remained unaffected by the temperature regime (Fig. 2). The mean survival of *P. regilla* was 58%, and not significantly affected by treatments (binomial GLM, *P* > 0.1). Numbers of *Daphnia* in mesocosms did not meaningfully predict the measured responses of *R. aurora* and *P. regilla*, and were dropped as covariates from final models. However, this was unsurprising, as none of the treatments significantly affected *Daphnia* numbers in tanks (ANOVA; *P* > 0.6 for all factors).

Throughout the experiment, we found strong evidence of inverse density dependence in the size and condition of tadpoles in mesocosms. In all models, the number of *R. aurora* survivors was a significant and strong predictor of *R. aurora* size (length and condition), with tadpoles growing larger at lower densities (ANCOVAs, *P* < 0.001).

**Figure 3:**
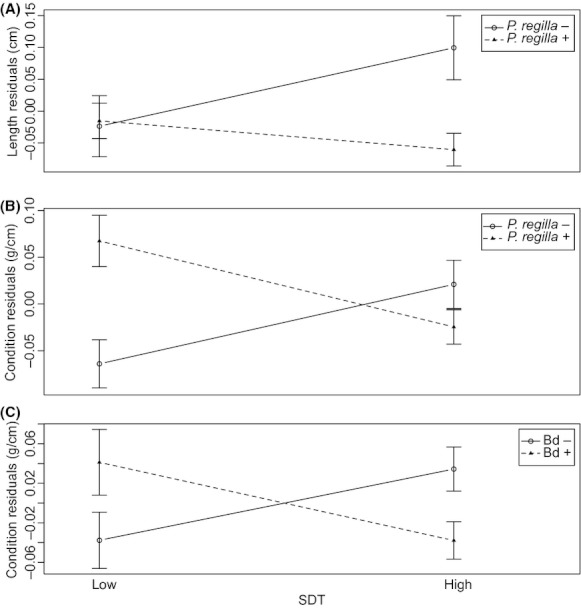
Significant two-way interactions from models testing effects of experimental factors on *Rana aurora* SVL (length) and condition (*mean* ± SE). *Data shown are predicted length and condition (mass / length) at metamorphosis using GAMs*. For consistency with the model fit, residuals of regressions of condition and length against surviving *R. aurora* density are shown. (A) In the absence of *P. regilla*, length increases with increasing SDT; in their presence it decreases (*P* = 0.009). (B) Body condition (mass shown) responds similar to length in the presence of *P. regilla* (*P* = 0.008). (C) *R. aurora* body condition decreases with increasing SDT when Bd is present (*P* = 0.003).

Temperature affected multiple aspects of the mesocosm tadpole community, including the response of *R. aurora* to the presence of *P. regilla*. Specifically, *R. aurora* length and condition both increased in the high SDT treatment in the absence of *P. regilla*, but decreased in the presence of *P. regilla* (ANCOVAs, *F*_1,43_ = 7.72, *P* = 0.009; *F*_1,39_ = 9.68, *P* = 0.003, for length and condition, respectively, Fig. 3).

Temperature also affected the growth response of *R. aurora* to Bd exposure. Although *R. aurora* exposed to high SDT when Bd was absent had improved condition, those exposed to high SDT in the presence of Bd had decreased condition, demonstrating temperature dependence in the response of *R. aurora* to Bd (Fig. 3C).

**Figure 4:**
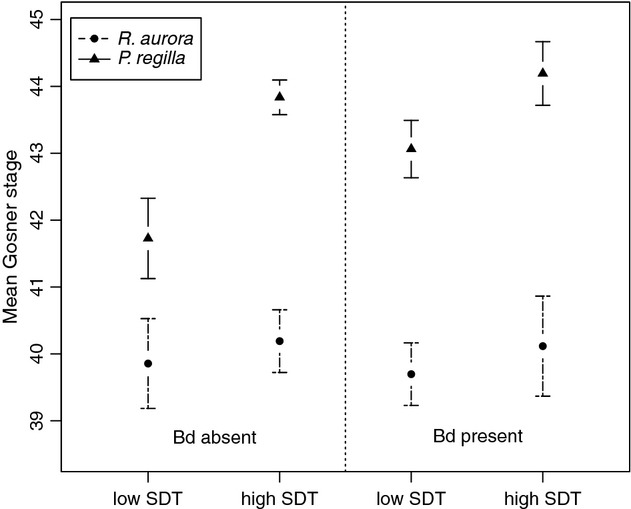
Mean and standard error of Gosner stages of *Rana aurora* and *Pseudacris regilla* in tanks containing both species, subjected to treatments of the presence and absence of Bd and low or high standard deviation of temperature. There are significant main effects of *Batrachochytrium dendrobatidis* and temperature on the difference in mean stages of the species (*P* = 0.029 and *P* = 0.007, respectively).

The response of *P. regilla* condition and length at metamorphosis were unaffected by treatments, after controlling for developmental stage (ANCOVA; *P* > 0.1 and *P* > 0.7 for all factors, respectively). However, whereas *R. aurora* development (Gosner stage at end of experiment) was not affected by treatments, *P. regilla* development increased significantly in response with treatments (Fig. 4). In general, *P. regilla* development stage was more advanced than that of *R. aurora* at the end of the experiment. Bd and high SDT increased this difference additively by 1.0 and 1.1 stages, respectively (ANCOVA; *F*_1,20_ = 9.17, *P* = 0.003, and *F*_1,20_ = 5.51, *P* = 0.03).

The prevalence of Bd infection in mesocosm tanks was notably low at the end of the experiment. Infection prevalence in *R. aurora*, detected using qPCR, ranged from 0 to 10% (mean ± SE = 7.9 ± 1.4%) in exposed tanks. Nineteen of 23 Bd-exposed tanks were detectably infected, whereas four tanks were not (one tank was not tested). Given the 70% initial infection rate of tadpoles used to introduce Bd to mesocosms, there was a 99.8% chance that Bd was added to each tank, suggesting that our inability to detect Bd in these tanks was due to either loss of infection from tadpoles, mortality of infected tadpoles or qPCR false-negatives, rather an initial absence of Bd. Analysis of Deviance indicated infection prevalence was not significantly affected by either SDT or *P. regilla* presence, although overall infection prevalence did tend to be slightly lower in the presence of *P. regilla* (ANODEV of binomial GLM; *P* = 0.5 and *P* = 0.06, respectively).

## Discussion

Our results demonstrate that increased variation in temperature (SDT) has numerous effects in tadpole communities. Many of these effects bear on our initial hypotheses and demonstrate the complexity that can be expected from species responses to interactions between temperature and disease (Lafferty 2009; Rohr et al. 2011). We hypothesized that increased SDT would increase the transmission and effects of Bd on *R. aurora*, the focal species in this study. In support of this, we found that exposure to Bd decreased the body condition of *R. aurora*, but only in the high SDT treatments. We did not, however, find an effect of SDT on Bd transmission, but the uniformly low infection prevalence at the end of this study made it difficult to detect treatment effects on transmission.

There was substantial evidence supporting our second hypothesis: that increased SDT can influence relationships between competing species. Responses of the length, condition, and survivorship of *R. aurora*, all indicated decreased performance of *R. aurora* in the presence of *P. regilla* at higher SDT. Likewise, *P. regilla* developed substantially faster at higher SDT, suggesting that *P. regilla* benefits relative to *R. aurora* at increased SDT. This is consistent with the apparent predilection of *P. regilla* for warmer, more extreme conditions than *R. aurora* in the wild (Brattstrom 1963).

There was little evidence supporting our third hypothesis, that interactions between Bd and SDT influence relative performance of competitors in mesocosms, in terms of effects of *P. regilla* presence on *R. aurora*. Although *R. aurora* survivorship was significantly influenced by a combination of increased SDT, Bd, and *P. regilla* presence, the effect was small. We did, however, find that both Bd and high SDT increased the performance of *P. regilla* relative to *R. aurora* in ponds. The additive effects of Bd and temperature can thus have effects in this system larger than either factor alone. This may indirectly influence species by shifting the relative performance of competing species.

Interestingly, *R. aurora* and *P. regilla* diverged in their response to treatments. *P. regilla* even appeared to benefit from the presence of Bd in mesocosms: they developed faster, but metamorphosed with the same length and condition in the presence of the pathogen, demonstrating that faster development did not come at a cost to size. Bd infection in tadpoles is limited to keratinized mouthparts, and infection may negatively affect feeding ability in susceptible tadpole species (Venesky et al. 2010), but *P. regilla* is apparently tolerant of Bd exposure and infection. Recently, *P. regilla* has even been implicated as an asymptomatic carrier of Bd (Reeder et al. 2012) that may readily disseminate infection to more susceptible species. Although little work has directly examined the effects of Bd exposure on *R. aurora*, closely related yellow-legged frogs (*Rana muscosa*) are highly susceptible to Bd infection and have undergone catastrophic population crashes attributed to chytridiomycosis (e.g., Wake and Vredenburg 2008). Altogether, *R. aurora* and *P. regilla* responses to Bd presence are consistent with earlier study suggesting that differences in the relative responses of species to Bd may lead to indirect community-level effects of the pathogen, which could even benefit more Bd-tolerant species (Bosch and Rincon 2008; Reeder et al. 2012)

The principal effect of the temperature manipulation in this study was to decrease daily SDT within tanks. A growing body of work has demonstrated or suggested important effects of temperature variance in the transmission and effects of parasites and pathogens, particularly those infecting or vectored by ectotherms (e.g., Raffel et al. 2006; Paaijmans et al. 2010; Rohr and Raffel 2010; Duncan et al. 2011; Murray et al. 2011). These effects could occur through multiple mechanisms. Higher temperature variation may increase the time spent at prohibitively high or low temperatures for either host or parasite, which has the potential to increase or decrease infection, depending on species response thresholds. This phenomenon has been demonstrated experimentally in microcosms (Duncan et al. 2011), and has been implicated as a factor limiting Bd infection in environmental suitability models (Murray et al. 2011). In addition, life history responses to fluctuations in temperature may influence disease dynamics. For instance, sudden drops in temperature trigger the release of infectious zoospores from Bd zoosporangia (Woodhams et al. 2008), setting the stage for complex interactions between host and pathogen in environments of fluctuating temperature. The magnitude of temperature variation and non-linearities in host and pathogen response to temperature may thus be expected to contribute to the extent to which these mechanisms affect host-pathogen systems. In this experiment, water temperatures never reached the 28°C upper threshold of temperature that Bd tolerates (Fig. 1; Piotrowski et al. 2004), and it is therefore unlikely that prohibitively high temperatures limited the growth of Bd.

The multiple effects we observed in tadpole communities exposed to different temperature treatments suggest a strong potential for direct and indirect effects of temperature variance on amphibian populations. Correlational studies increasingly implicate roles for climatic variance in the amphibian-Bd system, with diurnal temperature range a significant predictor of Central American amphibian declines that have been linked to chytridiomycosis (Rohr and Raffel 2010). Our results are thus consistent with the hypothesis of Rohr and Raffel (2010); that variation in temperature can be a driver of Bd's effects in host populations. Importantly, our results contribute that species differences in response to temperature variation can mediate its effects in ecological communities.

The final prevalence of Bd was low in tanks in this experiment, indicating little transmission. However, other factors might also account for the low observed prevalence, including mortality of infected individuals or clearance of Bd infection once acquired. Although there was some evidence that Bd contributed to mortality in this study in a three-way interaction between experimental factors, the effect was small and mortality was not consistently higher in Bd-present treatments. It is thus unlikely that a substantial number of Bd-infected individuals were lost due to mortality. Conversely, our data show a fitness cost associated with Bd in mesoscosms, at low infection frequency, with decreased metamorph condition in some circumstances. This is most consistent with a model of Bd infection in which exposed individuals acquire infection and eliminate it at physiological cost (Garner et al. 2009). Indeed, studies increasingly report the clearance of Bd from previously infected individuals (e.g., Garner et al. 2010; reviewed in Woodhams et al. 2011). Our sampling protocol may also have led us to underestimate the true prevalence of Bd in tanks somewhat. Sampling error (i.e., clipping uninfected toes from infected individuals in animals with low infection burdens (Boyle et al. 2004)), may have decreased the observed Bd prevalence and potentially obscured treatment effects on transmission in this study.

Altogether, infection prevalence was roughly representative of the 3–4% infection rate seen in *Rana* tadpoles of the Pacific Northwest (Pearl et al. 2009; P. Govindarajulu, unpubl. data). Another study running simultaneously on-site also observed per-capita effects of Bd at similarly low prevalence (Hamilton et al. 2012). These studies contribute to the growing body of literature that suggests effects of Bd on individuals that are not detectably infected, presumably due to transient infection and/or energetically costly immune defense (e.g., Garner et al. 2009). This may have implications for inferring Bd effects in wild populations based on prevalence in tadpoles, as Bd effects may not be directly coupled to prevalence.

*Rana aurora* are in decline in British Columbia (BC Ministry of Environment Conservation Data Centre), and their conservation status motivated this study to untangle the roles of potential drivers of decline. Using large-scale, multifactorial experiments has often been suggested to be necessary to adequately address ongoing amphibian declines as well as potential ecological consequences of climate change (e.g., Kilpatrick et al. 2010; Rohr et al. 2011). Herein, we manipulated the temperature and community composition of outdoor mesocosms and document modest but statistically significant effects on this declining species. The numerous effects we observe demonstrate that the response of amphibian tadpole systems to higher temperature variation is contingent on a complex of interacting factors, including the pathogens and competitors that are present.
